# Evaluation of Residual Right Ventricular Outflow Tract Obstruction After Pulmonary Valve-Sparing Repair of Tetralogy of Fallot: An Echocardiographic Study

**DOI:** 10.1177/21501351251336234

**Published:** 2025-05-14

**Authors:** Roberta Iacobelli, Priscilla Tifi, Gianluigi Perri, Zaccaria Ricci, Gianluca Brancaccio, Laura Ragni, Victoria d’Inzeo, Sergio Filippelli, Matteo Trezzi, Lorenzo Galletti

**Affiliations:** 1 Pediatric Cardiology, Clinical Area of Fetal and Cardiovascular Sciences, Bambino Gesù Children's Hospital, IRCCS, Rome, Italy; 2 Pediatric Cardiac Surgery, Clinical Area of Fetal and Cardiovascular Sciences, Bambino Gesù Children's Hospital, IRCCS, Rome, Italy; 3 Department of Emergency and Critical Care, Anesthesia and Pediatric Intensive Care Unit, Meyer Children's Hospital, IRCCS, Florence, Italy; 4 Department of Health Sciences, Section of Anesthesiology and Intensive Care, University of Florence, Florence, Italy

**Keywords:** pulmonary valve sparing repair, tetralogy of fallot, RVOT peak gradient, echocardiography

## Abstract

**Background:**

Pulmonary valve-sparing repair (PVSR) of Tetralogy of Fallot (TOF) provides good results in selected patients. However, recurrent right ventricular outflow tract obstruction (RVOTO) can occur requiring surgical revision. We sought to evaluate the course of RVOTO after PVSR by serial echocardiographic (ECHO) assessment.

**Methods:**

A retrospective analysis was conducted in patients who underwent PVSR of TOF at our institution. Demographic, anatomical, surgical and 2D-ECHO data were collected. The cohort was divided into two groups: no reintervention group (group 1) and reintervention group (group 2).

**Results:**

Ninety-one patients were included in this study; 13 patients (14%) required reintervention. Right ventricular outflow tract ECHO peak gradient was significantly higher in group 2 at intraoperative transesophageal ECHO (iTEE) (*P* = .009), at hospital discharge (*P* = .021), at six months follow-up (*P* = .0001) and 12 to 36 months follow-up (*P* = .0001). A more prevalent subvalvular stenosis was found in group 2 at six months (*P* = .0011) and 12 to 36 months follow-up (*P* = .00069). An RVOT ECHO peak gradient of 30 mm Hg at iTEE (*P* = .025) and discharge (*P* = .011) was statistically associated with the need for reintervention.

**Conclusions:**

Right ventricular outflow tract peak gradient was significantly higher in group 2 than in group 1 at iTEE, discharge and follow-up, with an ECHO peak gradient of 30 mm Hg being predictive of reintervention At follow-up, residual RVOTO was prevalent at the subvalvular level in group 2. This information will be useful in clinical decision-making for TOF patients undergoing pulmonary valve sparing repair.

## Introduction

Tetralogy of Fallot (TOF) is the most common cyanotic congenital cardiac defect, accounting for 10% of all congenital heart defects. Outcome after intracardiac repair is excellent. Pulmonary valve-sparing repair (PVSR) is currently recommended, when feasible, since pulmonary valve competence prevents chronic right ventricular volume overload and secondary late right ventricular dysfunction.^1–9^

However, recurrent right ventricular outflow tract obstruction (RVOTO) after PVSR can occur and may require surgical revision. Detection of residual gradient across the right ventricular outflow tract immediately after valve-sparing repair of TOF is not uncommon,^10^ but the acceptable threshold, and the risk factors predisposing to further recurrent obstruction requiring future surgical revision or percutaneous intervention are still a matter of debate.^6,11–13^

In the present study, we sought to identify risk factors for reintervention after PVSR by serial echocardiographic (ECHO) assessment as primary endpoint, and the recurrence of RVOTO as a secondary endpoint.

## Materials and Methods

### Study Population and Data Collection

From May 1999 to May 2023, a total of 536 patients affected by TOF underwent primary repair at our institution. Among these, all patients who underwent the valve-sparing technique for TOF at Bambino Gesù Hospital in Rome, Italy, with a minimum follow-up of 24 months after surgery, were retrospectively identified by our cardiac surgery database. The following patient characteristics were also retrospectively collected: age, body weight and body surface area (BSA) at surgery, sex, associated genetic syndromes, previous palliation history, and follow-up duration. Patients with pulmonary atresia or absent pulmonary valve were not evaluated in this study. Patients receiving a transannular patch (TAP) or being treated at an age > 24 months were also excluded from the analysis. Operation data, including surgical characteristics (such as the use of an infundibular patch or pulmonary patch) and the ratio of right ventricle pressure to aorta pressure after weaning off cardiopulmonary bypass at the end of surgery, were reviewed.The study conforms to the ethical principles of Good Clinical Practice, the Declaration of Helsinki, and the current Italian regulations. The IRB was notified and approved on August 10, 2024. The individual consent was waived for the retrospective nature of the analysis.

### Surgical Management

All complete repairs were performed after standard median sternotomy and cardiopulmonary bypass with moderate systemic hypothermia (28°-30°C). Antegrade Del Nido cardioplegia solution was used for myocardial protection. After right atriotomy, the obstructing muscle bundles were extensively divided and resected, and the VSD was exposed through the tricuspid valve. A heterologous pericardial patch was cut to the appropriate size and was sutured to the right side of the septum with continuous suture or with interrupted pledgetted sutures. The RVOT reconstruction was achieved through a transpulmonary approach or through the infundibulum (infundibulotomy). In the first case, the pulmonary valve was inspected, and a commissurotomy was performed if needed to increase the valve area. In cases where minimal or no commissural fusion was observed during gross examination, valve integrity was preserved to avoid PV regurgitation. Subsequently, a pericardial patch was used to reconstruct the pulmonary trunk before the bifurcation. When the transatrial/transpulmonary approach was insufficient for RVOT reconstruction, we performed a small ventriculotomy by a longitudinal incision below the pulmonary valve annulus with infundibulum muscle bundle resection and infundibular reconstruction with a pericardial patch. To obtain RVOT enlargement and to assess the adequacy of RVOT reconstruction, Hegar dilators were introduced through the RVOT to confirm adequacy of reconstruction based on normalized sizes according to BSA.

### Echocardiographic Evaluation

Preoperative and postoperative two-dimensional transthoracic and transesophageal echocardiograms (TTE and TEE) were reviewed and analyzed by two cardiologists blinded to clinical outcomes and to each other until the last follow-up. Preoperative PV anulus Z-score for all study patients were measured using the online Boston Z-Score calculator.^14^ The peak pressure gradient across the RVOT (RVOT peak gradient) was measured using continuous wave Doppler at intraoperative TEE, at discharge and during follow-up at TTE. If residual pulmonary gradient was present (RVOT peak gradient >16 mm Hg), the main level of residual pulmonary stenosis was carefully evaluated. If aliasing was visible below the pulmonary valve anulus and RVOT hypertrophy was present, the anatomic site of obstruction was considered subvalvular. On the other hand, if aliasing was visible at the level of the pulmonary valve, with restrictive opening of valvular leaflets, the main site of residual obstruction was considered as being at the anulus. At long-term follow-up, ECHO was also performed to assess recurrent RVOTO, pulmonary regurgitation (PR), tricuspid regurgitation, proximal pulmonary artery branch stenosis, right ventricular function, and/or dilation.

### Statistical Analysis

The cohort was divided into two groups: no reintervention group (group 1) and RVOT reintervention group (group 2) based on the necessity of reintervention, both surgical and/or percutaneous.

We conducted a univariate logistic regression analysis to assess if any association of predictive variables with the dichotomic outcome “reintervention” were present. With the same variables we conducted a multivariable logistic regression analysis (MLRA) in order to verify if the significant results remained independently associated with the outcome. A variation inflation factor below 3 was considered in order to exclude colinear variables. In this light two models were built: in one case RVOT peak gradient by ECHO at discharge was included in the MLRA and in the second intraoperative TEE (iTEE) RVOT peak gradient in order to assess their associations independently and to limit collinearity.

Chi-square analysis was used to compare discrete variables between the two groups, while continuous variables were compared by unpaired *t* test. Categorical analysis was conducted by chi square and Fisher exact testing. Early reoperation was defined as the need for reoperation within six months from surgery.

A *P* value <.05 was regarded as significant. Variables were expressed as the mean (±standard deviation) or median (range). The progression over time of RVOT peak gradient was studied with repeated measures using two-way ANOVA analysis.

The Receiver Operating Characteristic (ROC) curve was used to identify the RVOT peak gradient cutoff with the greatest sensitivity and sensibility to predict the need for reintervention.

We divided the patient sample into two groups based on the intraoperative and discharge RVOT peak gradient (RVOT peak gradient >30 mm Hg and RVOT peak gradient < 30 mm Hg). Differences between the two groups were assessed with the χ^2^ test for categorical variables. A *P* value <.05 was regarded as significant.

Additionally, a correlation between intraoperative mean RVOT peak gradient and right ventricle/left ventricle (RV/LV) pressure ratio ≥0.7 at the time of surgery was analyzed with *t* test analysis.Freedom from reoperation was assessed by Kaplan-Meier curve. Data analysis was performed using the SPSS software version 26 (IBM Corporation).

## Results

A total of 137 pediatric patients with TOF were identified as candidates for the valve sparing technique repair, according to anatomical characteristics.

The commonly used anatomical characteristics for PV annulus preservation included PV annulus size (Z value of less than −2.5 is the usual cutoff value) together with valve morphology (exclusion criteria are severely dysplastic PV with myxomatous/thickened pulmonary leaflets) and pulmonary artery branch size (indexed to BSA to exclude PA hypoplasia).

Thirty-six patients were excluded from the analysis because of loss to follow-up, eight patients were excluded for age at repair > 24 months, two patients initially considered candidates for valve-sparing technique, were also excluded due to inadequate relief of RVOTO and intraoperatively converted to TAP technique.

**Figure 1.**
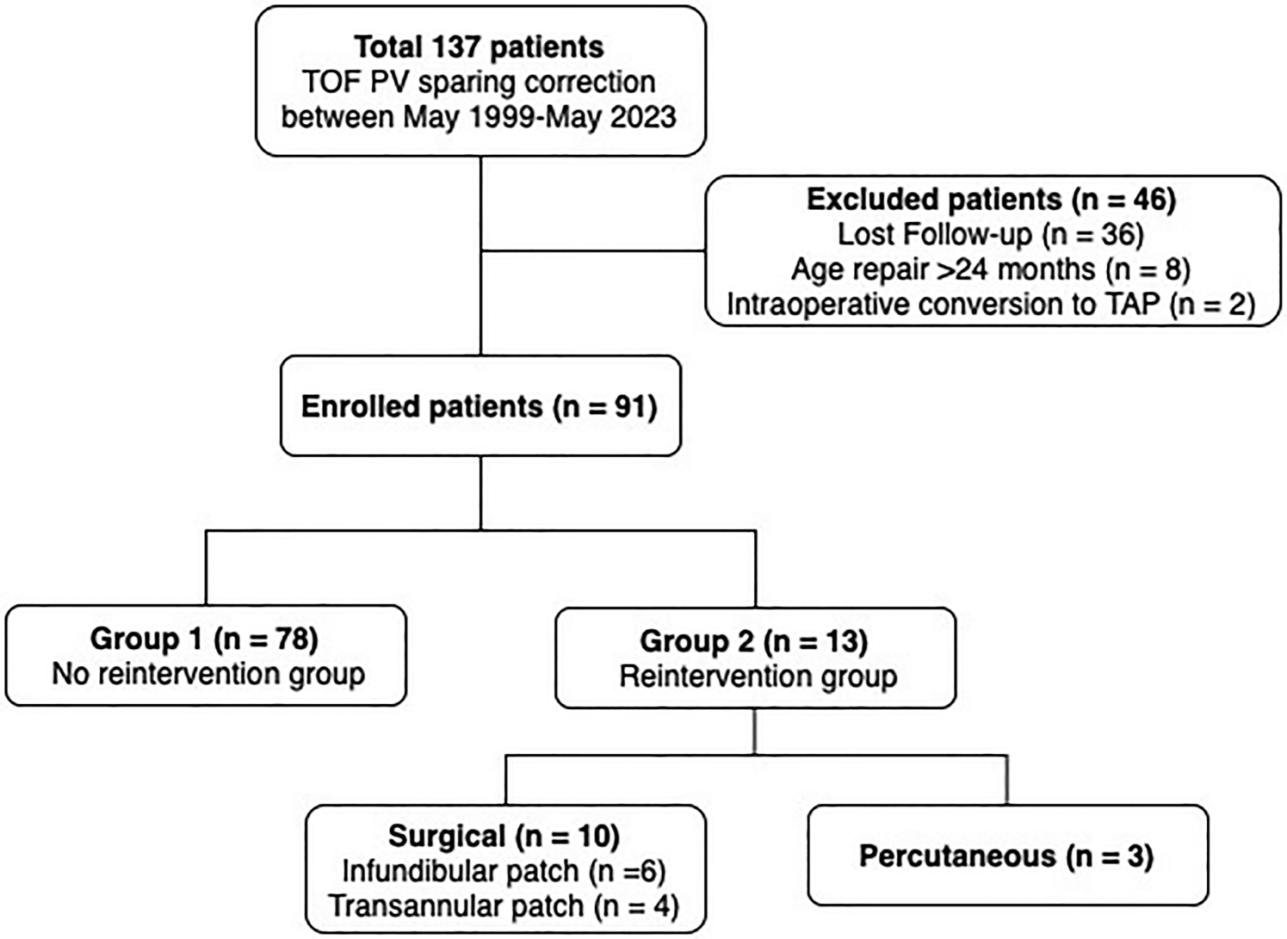
Flowchart of this study cohort. PV sparing, pulmonary valve-sparing; TOF, tetralogy of fallot.

As a result, 91 patients who received PVSR for TOF before the age of 24 months and who had a minimum of 24 months follow up were included in this study. The median follow-up duration was 115 months (range, 25-275) (Figure 1).

There were 56/91 (62%) males and the median age at time of surgery was 6.7 months (range, 0-24). The mean body weight was 6.6 kg (range 3.4-11.3 kg) mean BSA was 0.32 (range, 0.23-0.5), the mean PV anulus Z score was −1.3 (range, −2.9 to 0.8), 11/91 patients (12.1%) had a Z score < −2.5, while the majority of patients (70/91,76.9%) had a preoperative Z-score > −2.5 and <0. A genetic association was found in almost one-third of patients (31/91, 34%), most frequently Di George syndrome (19/91, 21%) followed by Down syndrome (14/91, 15.5%), Goldenhar and Vacterl syndrome (both 9/91, 9.1%), and Ano-rectal atresia (6/91, 6.1%), in 36/91, (39.5%) of patients other mutations were found. A total of 79/91 (87%) patients underwent total correction before 12 months of age; 12/91 (13%) patients underwent surgery between 12 and 24 months of age. A previous palliative procedure (Blalock-Taussig-Thomas [BTT] shunt) was performed in four of 91 patients (4%). All patients (except 4 who received a BTT shunt) did not present with a history of cyanotic spells, with a preoperative SpO2 between 85% and 95%. Coronary anomalies were present in four patients. Of 91 patients, 28 (30%) had a bicuspid pulmonary valve and 1 (1%) had a monocuspid pulmonary valve (Table 1).

**Table 1.** Baseline Population Characteristics at the Time of Surgery.

Total number of patients (n)	91
Males (n)	56 (62%)
Mean body weight (kg)	6.6 (3.4-11.3)
Mean BSA (kg/m^2^)	0.32 (0.23-0.5)
Genetic syndromes (n)	31 (34%)
Mean age (months)	6.7 (0-24)
Surgery before 12 months (n)	79 (87%)
Bicuspid pulmonary valve (n)	28 (30%)
Prevuious palliative procedures (n)	4 (4%)
Mean pulmonary valve Z score	−1.3 (−2.9 to 0.8)
Pulmonary valve Z score ≤ −2.5 (n)	11 (12.1%)
Pulmonary valve Z score > −2.5 ≤ 1.5 (n)	31 (34.1%)
Pulmonary valve Z score > 1.5− < 0 (n)	39 (42.8%)
Pulmonary valve Z score ≥ 0 (n)	10 (11.0%)

Abbreviation: BSA, body surface area.

The population was stratified into two groups (no reintervention group vs reintervention group) based on the need for any surgical procedure (RVOT infundibular patch or TAP) and interventional procedure (percutaneous pulmonary valvuloplasty, pulmonary artery stenting, or balloon angioplasty) related to RVOT/PA obstruction. There were no statistically significant differences in sex, body weight, age at surgery, associated syndromes, follow-up duration, palliative surgery, preoperative pulmonary valve annulus z-score, surgical technique (transatrial-transpulmonary or transventricular approach), and pressure ratio (RV/LV) in the operating room between the two groups (Table 2). Valve commissurotomy was performed in 38/91 (41.7%) of patients based on the valve anatomy observed during surgical inspection.

**Table 2.** Baseline Characteristics and Intraoperative Characteristics of the Two Groups.

Baseline characteristics of the two groups	No reintervention group (N = 78)	Reintervention group (N = 13)	*P* value
Sex (male)	48 (62%)	9 (70%)	.538
Body weight (kg)	5.08	5.57	.365
BSA (kg/m^2^)	0.32	0.33	.932
Syndromes	27 (34%)	4 (31%)	.786
Age at surgery (months)	6.85	5.92	.552
Bicuspid pulmonary valve (n)	25 (32%)	3 (23%)	.516
Palliative procedures (n)	3 (4%)	1 (8%)	.531
Preop pulmonary valve Z score	−1.32	−1.03	.305

Intraoperative characteristics of the two groups	No reintervention group (N = 78)	Reintervention group (N = 13)	*P* value
Infundibular patch	52 (67%)	8 (62%)	.718
Pulmonary patch	48 (62%)	9 (70%)	.596
PV commissurotomy	34 (43%)	4 (30%)	.386
Pressure ratio (right ventricle/aorta) in operating room	0.53	0.54	.983

Abbreviations: BSA, body surface area; PV, pulmonary valve.

A total of 13/91 (14%) patients required surgical or percutaneous reintervention. Among these, ten patients underwent surgical reintervention (six infundibular patch, four TAP), three patients underwent percutaneous reintervention (two percutaneous pulmonary valvuloplasty, one stenting of the left pulmonary artery branch). The median time to surgical reintervention was 40 months (range, 0-117), one patient required early reintervention (ie, reintervention <6 months from first surgery) with TAP before discharge for severe residual RVOTO after valve-sparing surgery (Table 3).

**Table 3.** Type and Timing of Reintervention in 13 Patients Requiring Relief of Residual Right Ventricular Outflow Tract.

Surgical reintervention (10 pts)	Timing 0-6 months (pts)	Timing 1-3 years (pts)	Timing 3-5 years (pts)	Timing 5-10 years (pts)
Infundibular patch (6 pts)	0	1	4	1
Transannular patch (4 pts)	1	1	2	0
Percutaneous reintervention (3 pts)	Timing 0-6 months (pts)	Timing 1-3 years (pts)	Timing 3-5 years (pts)	Timing 5-10 years (pts)
Ballon PV valvuloplasty (2 pts)	0	1	0	1
LPA stenting (1 pt)	0	1	0	0

Abbreviations: LPA, left pulmonary artery; PV, pulmonary valve.

**Figure 2.**
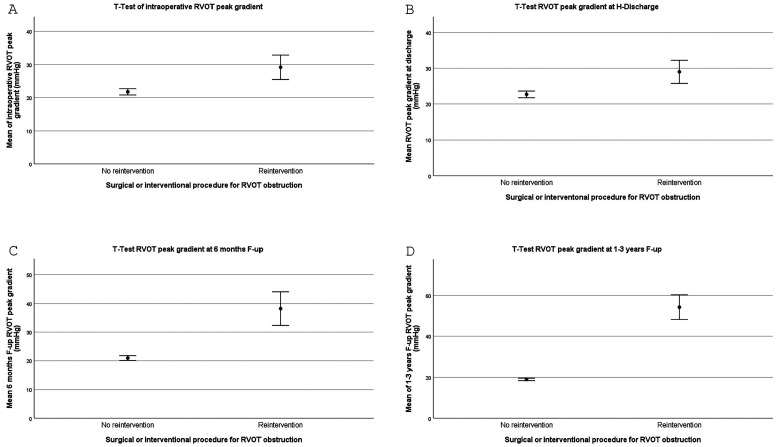
(A) *T* test of intraoperative RVOT echocardiographic (ECHO) peak gradient ± standard error (SE); (B) *T* test of hospital discharge RVOT ECHO peak gradient ± standard error (SE); (C) *T* test of six months follow-up RVOT ECHO peak gradient ± standard error (SE); (D) *T* test of 1 to 3 years (12-36 months) RVOT ECHO peak gradient ± standard error (SE). RVOT, right ventricular outflow tract.

We found that RVOT peak gradient was significantly higher in the reintervention group versus no reintervention group as measured by TEE at intraoperative evaluation (29.14 ± 3.69 vs 21.71 ± 0.95, *P* = .009), at discharge (29.00 ± 3.23 vs 22.68 ± 0.93, *P* = .021), at six months follow-up (38.25 ± 5.89 vs 20.93 ± 0.84, *P* = .0001), and at 12 to 36 months follow up (54.25 ± 6.04 vs 19.03 ± 0.59, *P* = .0001) (Table 4; Figure 2A-D).

**Table 4.** Mean RVOT Peak Gradient in the Two Groups at Intraoperative Evaluation, at Hospital Discharge, at Six Months Follow-up and at 12 to 36 Months Follow-up.

Mean RVOT peak gradient (mm Hg)	No reintervention group	Reintervention group	*P* value
Intraoperative	21.71 ± 0.95	29.14 ± 3.69	.009*
At hospital discharge	22.68 ± 0.93	29.00 ± 3.23	.021*
At 6 months follow up	20.93 ± 0.84	38.25 ± 5.89	.0001*
At 12-36 months follow up	19.03 ± 0.59	54.25 ± 6.04	.0001*

Abbreviation: RVOT, right ventricular outflow tract. *Statistical significance.

A univariate analysis was conducted with predictors of reintervention (ie, PV Z-score, age at repair, intraoperative ECHO RVOT peak gradient and at discharge, associated syndrome, type of surgery, postoperative RV/LV pressure ratio, previous palliation) showed that the only significant variable associated with reontervention were RVOT peak gradients. This result was further confirmed at multivariate regression analysis in which the two models including RVOT peak gradient by ECHO at discharge and iTEE RVOT peak gradient showed that they were both independently associated with reintervention rate (*P* = .02 and .04, respectively) (Tables 5 and 6).

**Table 5.** Univariate and Multivariate Analysis of First Model (RVOT ECHO Peak Gradient at Discharge).

	Univariate			Multivariate		
Variable	OR	95% CI	*P* value	OR	95% CI	*P* value
Intercept	-	-	-	0.04	0.00-1.66	.09
PV Z-score	1.40	0.73-2.72	.30	2.49	0.90-8.38	.09
Age at repair (month)	0.96	0.82-1.07	.55	0.94	0.75-1.12	.59
RVOT ECHO peak gradient at discharge	**1** **.** **07**	**1.00-1.15**	.**03**	**1**.**11**	**1.01-1.23**	.**02**
Associated syndrome	0.83	0.21-2.84	.78	0.95	0.11-5.49	.96
Infundibular patch	0.8	0.24-2.87	.71	1.91	0.34-13.91	.47
Postoperative RV/LV pressure ratio	1.00	0.98-1.02	.71	0.99	0.95-1.02	.57
Previous palliation	2.08	0.09-17.86	.53	1.62	0.02-54.52	.79

Abbreviations: CI, confidence interval; ECHO, echocardiography; LV, left ventricle; OR, odds ratio; PV, pulmonary valve; RV, right ventricle; RVOT, right ventricular outflow tract.

**Table 6.** Univariate and Multivariate Analysis of Second Model (Intraoperative RVOT Peak Gradient).

	Univariate			Multivariate		
Variable	OR	95% CI (profile likelihood)	*P* value	OR	95% CI (profile likelihood)	*P* value
Intercept	—	—	—	0.10	0.00-25.25	.390
PV Z-score				3.03	0.68-22.17	.184
Age at repair (month)				0.69	0.35-1.04	.174
Associated syndrome				1.73	0.06-31.19	.704
Intraoperative RVOT peak gradient	**1.15**	**1.03-1.33**	**.02**	**1**.**17**	**1.00-1.46**	.**04**
Infundibular patch				2.11	0.16-46.28	.57
Postoperative RV/LV pressure ratio				0.98	0.92-1.02	.43
Previous palliation				2.36	0.00-302.8	.72

Abbreviations: CI, confidence interval; LV, left ventricle; OR, odds ratio; PV, pulmonary valve; RV, right ventricle; RVOT, right ventricular outflow tract.

**Figure 3.**
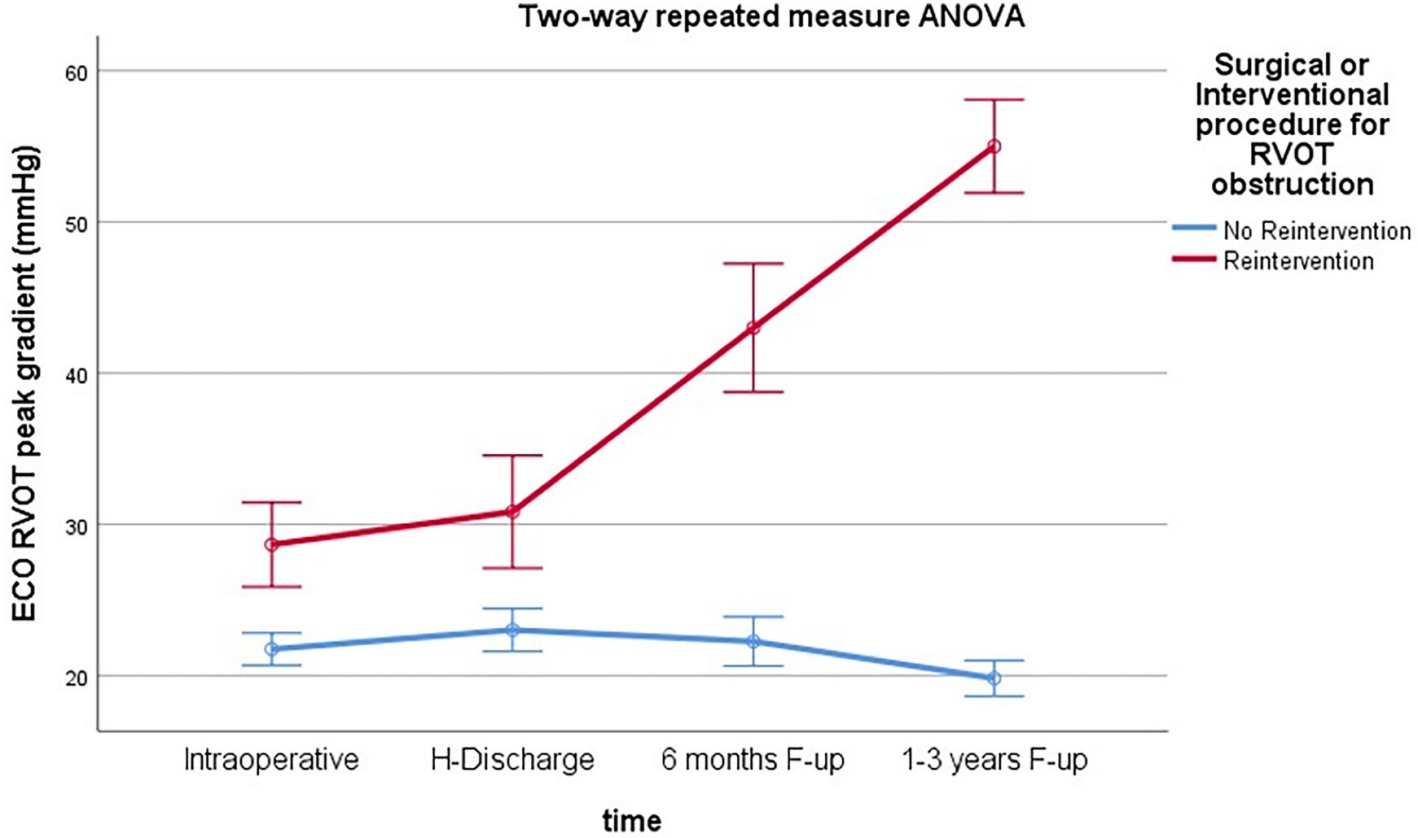
Two-way repeated measure ANOVA test. Graph of RVOT peak gradient progression over time. RVOT, right ventricular outflow tract.

The two-way repeated measure ANOVA test confirmed the significant increase in RVOT peak gradient in time in the reintervention group and statistical difference between the two groups (Figure 3).

**Figure 4.**
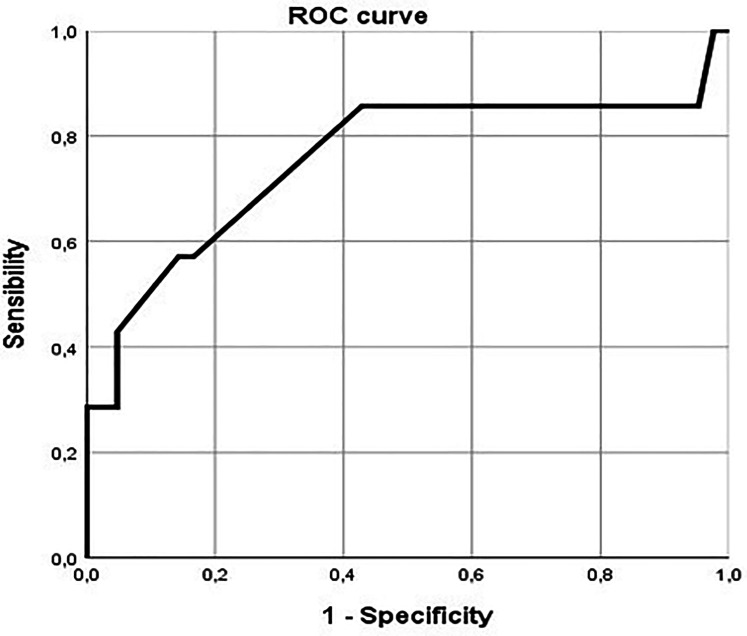
ROC curve showing sensibility and specificity of intraoperative RVOT echocardiographic peak gradient of 30 mm Hg. RVOT, right ventricular outflow tract.

The ROC curve showed an area under curve of 0.757, acceptable discrimination in diagnostic accuracy of RVOT peak gradient in predicting need for reintervention (Figure 4). The cutoff of 30 mm Hg has a sensibility of 57.1% and a specificity of 85.7% in identifying need for reintervention.

Additional analysis was performed dividing the population in two groups based on the intraoperative and discharge RVOT peak gradient (RVOT peak gradient >30 mm Hg and RVOT peak gradient < 30 mm Hg) to see whether there was a correlation between residual RVOT intraoperative obstruction and need for future reintervention.

Chi-square analysis showed that a value of RVOT peak gradient ≥30 mm Hg at intraoperative evaluation (*P* = .025) and at discharge (*P* = .011) was statistically associated with the need for reintervention.

**Figure 5.**
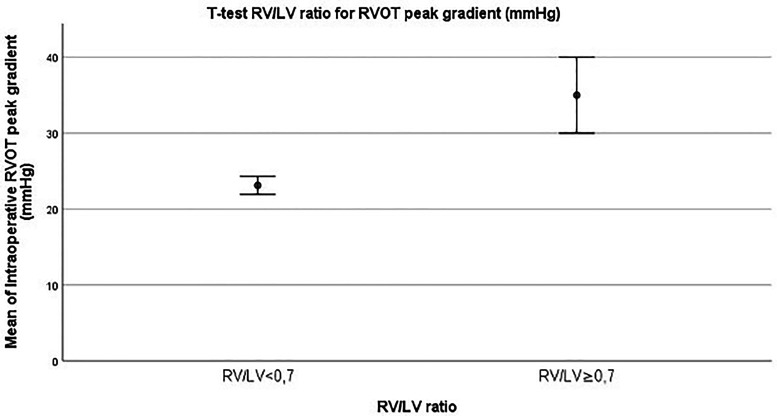
*T* test of intraoperative RV/LV pressure ratio and RVOT peak gradient (mm Hg) ± standard error (SE). LV, left ventricle; RV, right ventricle; RVOT, right ventricular outflow tract.

Additionally, a correlation between intraoperative mean RVOT peak gradient and RV/LV pressure ratio ≥0.70 at time of surgery was analyzed with *t* test analysis. We found that there was a statistically significant correlation between RV/LV pressure ratio and mean intraoperative RVOT peak gradient (23.12 mm Hg ± 1.20 vs 35 ± 5; *P* = .024) (Figure 5).

Finally, a difference in the anatomic type of residual RVOT obstruction (subvalvular vs non subvalvular) with a peak gradient >16 mm Hg at discharge and during FU was analyzed by χ^2^ analysis among the two groups (reintervention vs no reintervention). We found that although there were no statistically significant differences at discharge (*P* = .549), there was a more prevalent subvalvular stenosis in the reintervention group at six months and 12 to 36 months follow up (*P* = .0009 at six months and *P* = .0005 at 12-36 months, respectively).

**Figure 6.**
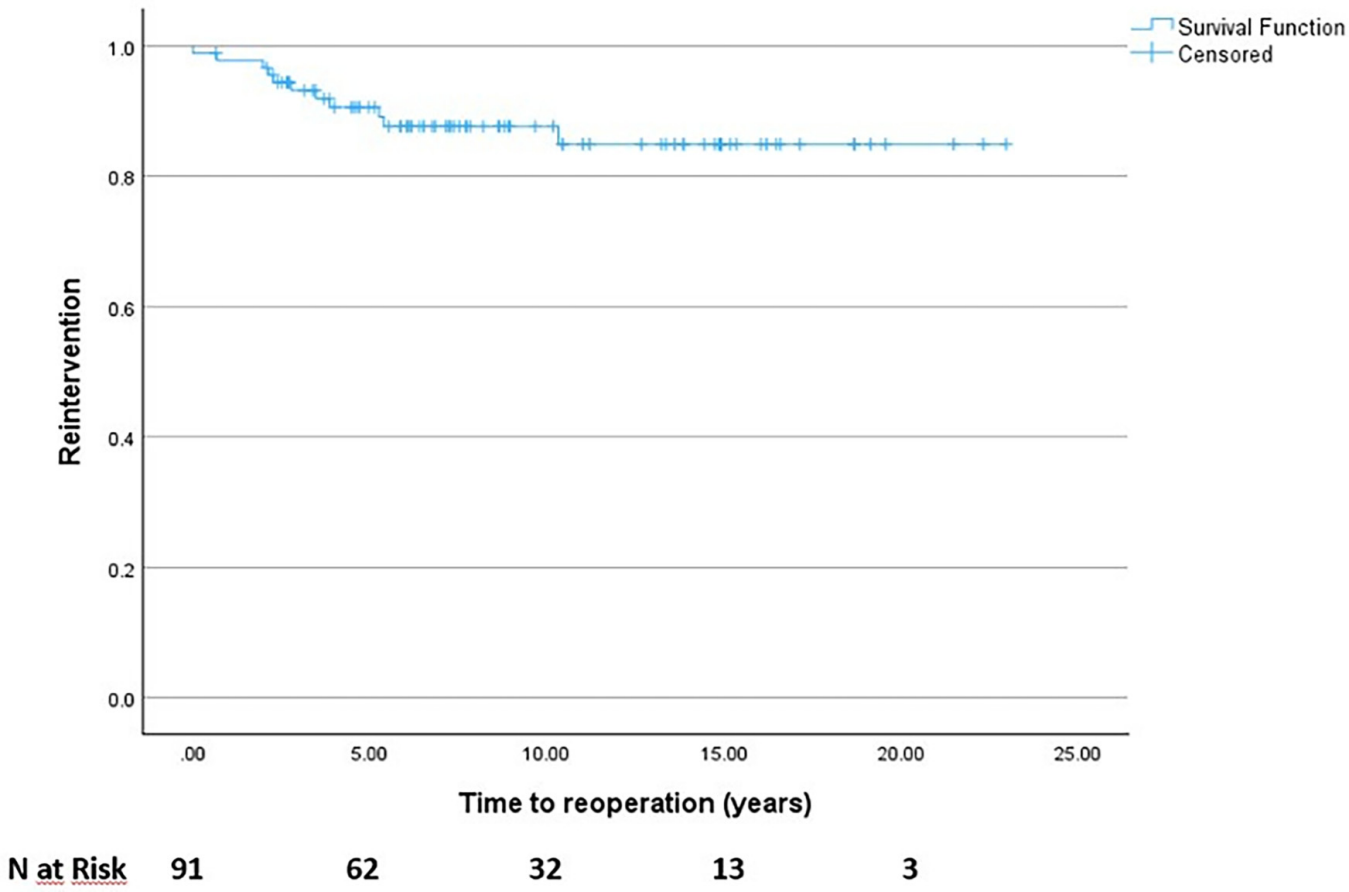
Freedom from reintervention (Kaplan-Meier curve) with number of patients at risk.

Kaplan-Meier survival curves were used to calculate freedom from reintervention; from the analysis we observed that 86% of our patient population were free from reintervention at a medium follow up of 115 months with a major rate of reintervention occurring between 1 to 3 years and 3 to 5 years from the primary surgical repair procedure (Figure 6).

## Discussion

Despite decades of experience in TOF treatment with positive outcomes, there is still debate about the best management approaches, with significant differences in practice and many opportunities for further research to enhance care.^1^

According to Blais and colleagues,^15^ patients that underwent a pulmonary valve sparing procedure had increased 30-year survival, fewer cardiovascular reinterventions, and fewer pulmonary valve replacements compared with those who underwent TAP repair, even in the presence of significant residual pulmonary stenosis. Boni and colleagues^16^ confirmed the importance of preserving the integrity of the pulmonary valve for better long-term prognosis, despite the risk of residual stenosis, because chronic volume overload after TAP repair of TOF is associated with greater late morbidity and mortality.

As previously mentioned, it is not uncommon to detect a residual gradient across the RVOT immediately after TOF valve-sparing repair.

Many institutions and surgeons have their own thresholds for when to consider reintervention. This is often done by performing a TAP procedure if the issue is fixed obstruction at the level of the PV annulus. Although such thresholds are often mentioned in various articles, the supporting evidence is limited and is still a matter of debate. In the literature, numerous variables are considered to determine the risk of reintervention, with intraoperative RV/LV ventricle pressure ratio and RVOT peak gradient being two of the most commonly used. In past years, Hennein identified both RV/LV pressure ratio >0.75 and RVOT obstruction at discharge of 40 mm Hg as risk factors for reintervention.^17^ More recently, some researchers have proposed different criteria for performing a TAP. Bove et al^18^ suggested that an RV/LV ratio of less than 0.8 is acceptable, while a higher ratio is associated with risk of reoperation. Lozano-Balseiro et al^19^ suggested a threshold of 30 mm Hg in the RVOT as an indication to perform a TAP. Choi et al^20^ suggested that a cutoff of RV/LV pressure ratio greater than 0.8 or an RVOT peak gradient of 40 mm Hg is an indication to go back on pump and perform a TAP. Boni and colleagues included patients with RV pressure >70% of LV pressure post repair and found that the ratio of RV pressure relative to LV pressure decreased significantly by 28% for those with RV pressure >70% of LV pressure, whereas those with RV pressure <70% of LV pressure decreased non significantly by 12% after a median of 33 months.^16^ In a recent article, Kim and colleagues concluded that acceptable immediate residual pulmonary stenosis (less than 45 mm Hg) in corrected TOF patients can progress, and a higher pressure gradient than 26.8 mm Hg and stenosis that originated from the subvalvular area were significant factors in progression.^21^ Lastly, in a large cohort of TOF patients who underwent both PVS and TAP techniques, Ishigami et al found that those with a postoperative peak RVOT gradient of 25 mm Hg or greater had a significantly increased risk for reoperation for any cause. Preserving the pulmonary valve reduces the risk of requiring a pulmonary valve replacement, but it also increases the rate of reoperation for RVOTO; no differences were observed in terms of total reoperation rates between PVS and TAP repair.^22^

In our study, we aimed to evaluate the course of RVOTO after pulmonary valve-sparing surgical repair and identify risk factors for progression and subsequent need for reintervention by serial ECHO assessment. At multivariate regression analysis, we found that RVOT peak gradient ECHO at discharge and iTEE RVOT peak gradient were independentely associated with reintervention (*P* = .02 and .04, respectively). Furthermore, the mean RVOT ECHO peak gradient was significantly higher in the reintervention group with a consistent growth in the mean pressure gradient over time as measured by TEE at: intraoperative evaluation (29.14 ± 3.69 vs 21.71 ± 0.95, *P* = .009), discharge (29.00 ± 3.23 vs 22.68 ± 0.93, *P* = .021), six months follow-up (38.25 ± 5.89 vs 20.93 ± 0.84, *P* = .0001), and 12 to 36 months follow up (54.25 ± 6.04 vs 19.03 ± 0.59, *P* = .0001). We also found that RVOT ECHO peak gradient ≥30 mm Hg at intraoperative evaluation and at discharge was predictive of reintervention, with a sensitivity of 57.1% and a specificity of 85.7% in identifying the need for reintervention. This value is consistent with the literature and with what is proposed in other studies.^19^

Furthermore, we also focused on characterizing the anatomy of the RVOT distinguishing cases in which the dominant level of obstruction was at: infundibular level or subvalvular level; valvular level, due to PV annulus hypoplasia or valvular leaflet dysplasia. Despite ECHO accurate evaluation of PV Z-score, morphological evaluation of pulmonary valve (number and mobility of leaflets, commissures) in the preoperative period is fundamental to predict the risk of persistence of a valvular additional component of stenosis.^20^ The annular Z-score is a measure of the annulus and does not necessarily identify the effective orifice of the valve: a normal Z-score may be associated with a small effective orifice with subvalvular stenosis.^4^ This could correlate with a progressive growth of muscle bundles in patients with residual valvular RVOTO that can progress over time in a subvalvular obstructive component.

While at hospital discharge the degree of RVOT peak gradient was mainly due to a valvular component in both groups, we observed the development of a mainly subvalvular component at six months and 12 to 36 months follow up (*P* = .0009 at six months and *P* = .0005 at 12-36 months, respectively) in the group that required later reintervention. Additionally, we focused on the relationship between the RV/LV pressure ratio and RVOT obstruction. We found a statistically significant correlation between RV/LV pressure >0.7 and RVOT peak gradient, with higher values of mean RVOT peak gradient in the group with RV/LV ratio >0.7 (23.12 mm Hg ± 1.20 vs 35 ± 5; *P* = .024). Finally, according to our follow up data, 78/91 (86%) of our patient population were free from reintervention at a medium follow up of 115 months with a major rate of reintervention occurring between 1 to 3 years and 3 to 5 years from the primary surgical repair procedure.

While this study was not intended to give recommendations on when it is appropriate to switch intraoperatively from a valve sparing technique to TAP,^23^ since the latter is associated with future development of RV dysfunction due to severe PR and need for PV replacement, from our data we mean to emphasize that in PVSR an intraoperative and/or discharge RVOT ECHO peak gradient > 30 mm Hg is frequently associated with the need for reintervention. This value is lower than what was reported in other studies^17^ and consistent with recent literature suggesting more restrictive cutoffs.^22^

## Study Limitations

There are several potential limitations to our study, including its retrospective design and the relatively small number of patients. Although ECHO studies were carried out by experienced professionals, measurement error was inevitable; Continuous Wave (CW) Doppler alignment on the RVOT is sometimes challenging because of the anterior aspect of the RVOT during TEE evaluation. Furthermore, hemodynamic conditions during surgery, such as filling pressures or systemic pressure, as well as the administration of inotropic drugs (not desirable after weaning from cardiopulmonary bypass) could be different in the patient population, but these data were not available in our study.

Again, we were not able to create PV z-score classes with our distribution, in contrast with the values reported by two centers that focused more on PV regurgitation^24^ and in intraoperative balloon valve dilatation,^25^ respectively.

We did not analyze the mean PV Z-score after surgery. In our study, balloon PV valvuloplasty was performed in only three patients during follow up, while surgical revision was necessary for ten patients, primarly due to the subvalvar component of the RVOTO. Further analysis is needed to evaluate the role of transcatheter procedures in patients with residual obstruction at the PV level.

To overcome these intrinsic limitations of our study,although it was carried out in a tertiary center by highly specialized pediatric cardiologists and with relatively restrictive inclusion and exclusion criteria, well-designed, prospective, long-term studies across multiple centers are necessary to obtain reliable and precise outcomes.

## Conclusion

In a population of pediatric patients with TOF undergoing a pulmonary valve sparing repair, RVOT peak gradient as measured by ECHO was significantly higher in the reintervention group (group 2) than in the no reintervention group (group 1) at intraoperative evaluation, at discharge, and during follow-up. A peak ECHO gradient of 30 mm Hg or more at intraoperative TEE or at discharge was predictive of the need for reintervention. Anatomic subvalvular type of residual RVOTO was more prevalent in group 2 than in group 1 during follow-up; 78/91 (86%) of our patients were free from reintervention at a medium follow up of 115 months, with the major incidence of reintervention occurring in the first five years from the primary surgical repair. This information should be useful for intraoperative decision-making and clinical follow-up after pulmonary valve sparing repair of TOF.

## Abbreviations

BSA: body surface area
BTT: Blalock-Taussig-Thomas
ECHO: echocardiography
iTEE: intraoperative transesophageal echocardiography
MLRA: multivariable logistic regression analysis
PR: pulmonary regurgitation
PVSR: pulmonary valve-sparing repair
RVOTO: right ventricular outflow tract obstruction
TAP: transannular patch
TOF: Tetralogy of Fallot
